# Observation of Electron Spin Dynamics on Hundreds-of-Microseconds Timescales in High-Field Magic Angle Spinning EPR

**DOI:** 10.1021/acs.jpclett.6c02108

**Published:** 2026-09-19

**Authors:** Sudipta Khamrui, Orit Nir-Arad, Frederic Mentink-Vigier, Alexander B. Fialkov, Nurit Manukovsky, Ilia Kaminker

**Affiliations:** † School of Chemistry, 26745 Tel-Aviv University, Tel-Aviv 6997801, Israel; ‡ National High Magnetic Field Laboratory, Florida State University, Tallahassee, Florida 32310, United States; § Department of Chemistry and Biochemistry, Florida State University, Tallahassee, Florida 32306, United States

## Abstract

Dynamic nuclear polarization (DNP) efficiency critically depends on electron–nuclear polarization transfer, necessitating a thorough understanding of electron spin dynamics under DNP-relevant conditions, namely, high magnetic fields and magic angle spinning (MAS). While high-field electron paramagnetic resonance (EPR) under MAS is the only tool capable of probing these dynamics, its application has been limited by technological constraints and methodological gaps, leaving the microscopic behavior of electron spin packets under MAS largely unexplored experimentally. Here we demonstrate the first rotor-synchronized three-pulse stimulated echo MAS-EPR experiments at 6.9 T with spinning frequencies up to 37 kHz. This approach enables the characterization of electron spin dynamics on timescales of hundreds of microseconds under MAS, revealing its strong dependence on both EPR line anisotropy and MAS rate. We further validated our experimental findings with time-domain numerical simulations. This work establishes a robust methodology for investigating electron spin dynamics in MAS-DNP on the experimentally relevant timescales.

## Graphical abstract

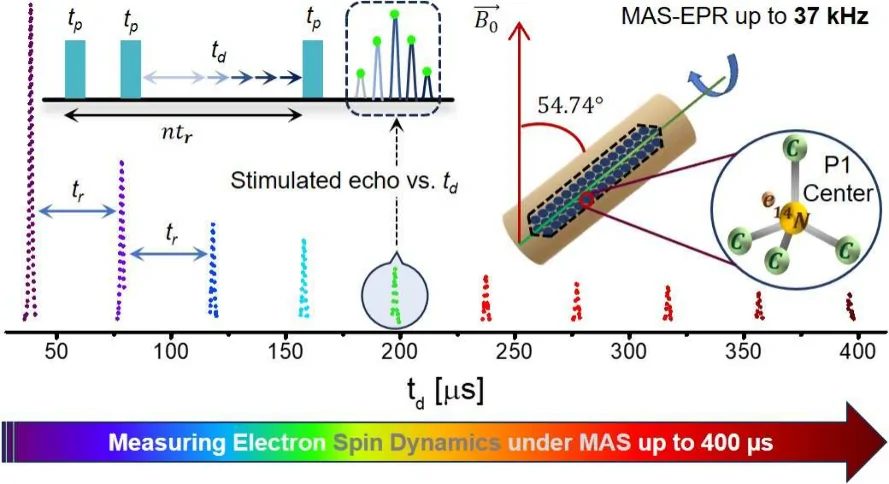

Electron paramagnetic resonance (EPR) spectroscopy is essential for probing the electronic structure and dynamics of paramagnetic species. While typical EPR experiments operate at 0.3–1.5 T, high-field EPR (>3 T) offers distinct advantages.^1–3^ The increased Zeeman interaction resolves species with similar *g*-tensors and narrows the line width of central transitions in half-integer high-spin systems. High fields are also critical for experiments involving both electron and nuclear spins. For example, in electron–nuclear double resonance (ENDOR), the greater separation of nuclear Larmor frequencies at high fields simplifies signal assignment.^4–6^ Similarly, high-field EPR is vital for studying dynamic nuclear polarization (DNP). Since NMR sensitivity and resolution improve with magnetic field strength, high-field EPR is necessary for the investigation of polarization transfer that enhances NMR signals.^7–14^

In DNP, irradiation near the electron Larmor frequency with millimeter waves (mm-Waves) transfers polarization from unpaired electrons to nuclear spins, creating nuclear hyperpolarization.^15–17^ While the theoretical mechanisms of this process are well-understood for simple mono- and biradical polarizing agents,^18–27^ complex spin systems remain challenging. This is particularly true for high-spin species^8,28,29^ or samples with inhomogeneous spatial distributions,^30^ where the lack of experimental methods to probe electron spin dynamics hinders the optimization of MAS-DNP. Rational DNP design, therefore, requires knowledge of electron spin dynamics, typically obtained via pulse EPR.

Historically, static pulse EPR has aided our understanding of DNP mechanisms.^31–43^ However, contemporary DNP is often performed at high fields (> 5 T) under magic angle spinning (MAS),^22,44–46^ where the sample rotates rapidly around an axis tilted by θ_m_ ∼ 54.74° relative to the external magnetic field. DNP performance is known to depend on measurement conditions,^31,39–43^ rendering static EPR experiments of limited value in capturing the relevant spin dynamics. Consequently, developing a toolkit of high-field pulsed EPR experiments under MAS is essential. A major challenge is to effectively couple mm-Waves to the rotating sample, a problem exacerbated by the power limitations of mm-Wave sources suitable for pulsed EPR. Reports of MAS-EPR in the literature are scarce. Early attempts in 1997 and 2000 were conducted by the Spiess group at only 0.3 T field, aiming to improve EPR spectral resolution^47,48^ and were not pursued further. A recent gyrotron-powered continuous-wave MAS-EPR experiment was reported by Barnes’s group at 7 T.^49^ We recently demonstrated the first high-field pulsed MAS-EPR at 6.9 T using a spherical rotor, achieving spinning frequencies up to 4 kHz.^50^ However, two-pulse echo experiments only allowed us to observe electron spin dynamics over a few microseconds. Since DNP dynamics typically occur over longer timescales and at higher spinning rates, new experimental methodologies are required.

In this Letter, we present the first rotor-synchronized three-pulse stimulated echo MAS-EPR experiments at spinning frequencies up to 37 kHz. This approach enables the observation of electron spin dynamics over hundreds of microseconds under MAS conditions. We further characterize how these dynamics depend on MAS rates and EPR line anisotropy, showing that our numerical simulations agree well with the experimental findings.

We used a microdiamond powder sample with a high concentration (∼120 ppm or nearly 35.1 mM) of P1 defects (substitutional nitrogen centers). This system consists of an unpaired electron spin *S* = 1/2 coupled to a nuclear spin *I* = 1 via hyperfine interaction. The static EPR spectrum, recorded at 6.9 T using two-pulse echo frequency-swept measurements, is characteristic of P1 centers. As shown in Figure 1a (black trace), the spectrum possesses three allowed EPR transitions. The central peak (*m*_*I*_ = 0) has a narrow and symmetric lineshape because it is unaffected by the hyperfine interaction. Its resonance frequency is nearly isotropic due to minimal *g* anisotropy (*g*_⊥_ = 2.00220 ± 0.00001, *g*_∥_ = 2.00218 ± 0.00001).^51^ In contrast, the outer peaks (*m*_*I*_ = ±1) are broadened by the anisotropic hyperfine interaction (*A*_⊥_ = 81.3 MHz, *A*_∥_ = 114 MHz). For these transitions, the resonance frequency of each spin packet depends strongly on the relative orientation of the electron–^14^N hyperfine interaction tensor with respect to the external magnetic field. Additionally, a broad EPR signal is visible between the *m*_*I*_ = 0 and *m*_*I*_ = ±1 lines. This signal is attributed to P1 center clusters and is dominated by exchange and dipolar interactions between the cluster members.^51,52^

**Figure 1:**
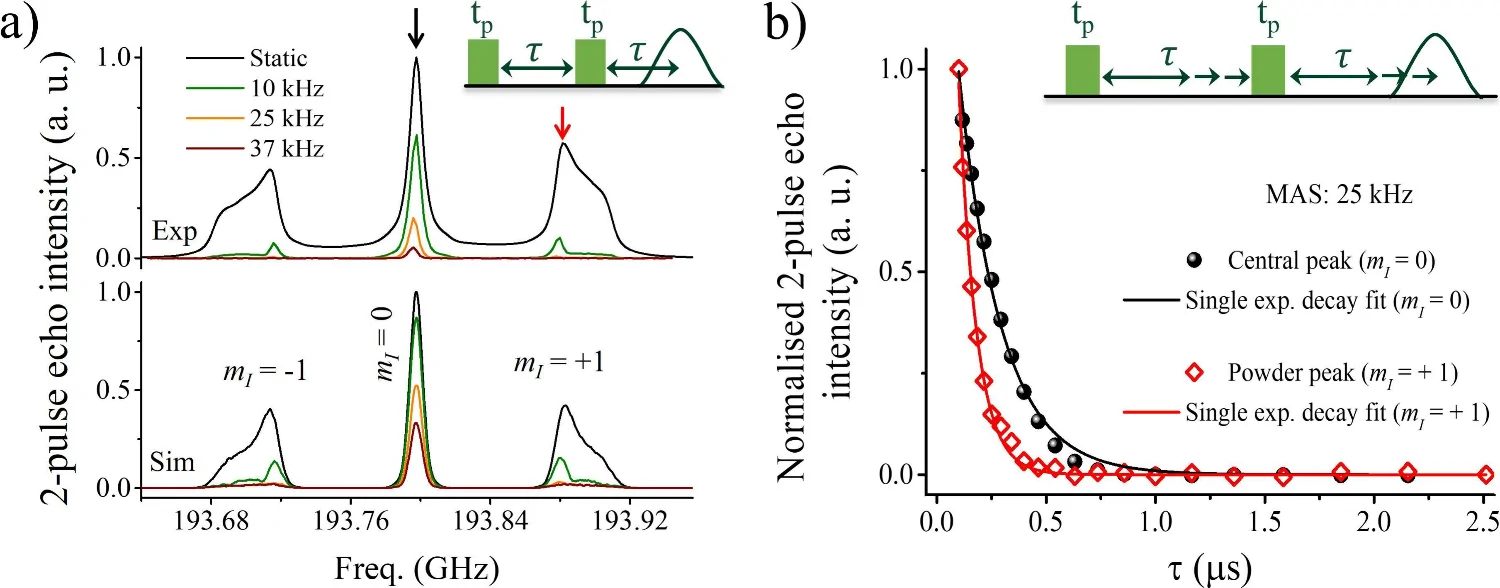
Two-pulse echo-detected EPR measurements at room temperature. (a) Frequency-swept spectra under static conditions and at different MAS frequencies: (top) experimental; (bottom) simulated. The intensities are normalized to the static spectrum. The inset displays the pulse sequence. (b) Two-pulse echo decay traces at the central peak (black) and the maximum of the right powder line (red) measured at 25 kHz MAS. Solid lines are single-exponential fits to the experimental data. The black and red down arrows in (a) indicate the positions where the decays in (b) were recorded. Pulse sequence parameters: *t*_p_ = 400 ns, τ = 300 ns.

Under MAS, the rapid change in the orientation of magnetic tensors causes the resonance frequencies of spin packets to vary during the pulse sequence. In a two-pulse echo experiment with selective pulses, this variation prevents effective refocusing: the spin packets excited by the first mm-Wave pulse at a specific phase and frequency cannot be properly refocused due to MAS-induced phase and frequency shifts. This results in reduced EPR echo intensity, a phenomenon known as MAS-induced spin dephasing.^50^ The extent of this dephasing depends on the overall anisotropy of the EPR line and the available mm-Wave power. Since the central peak (*m*_*I*_ = 0) has minimal *g*-anisotropy while the outer peaks (*m*_*I*_ = ±1) are dominated by hyperfine anisotropy, the dephasing effect is expected to be more pronounced for the outer peaks.

The top panel in Figure 1a illustrates this behavior by comparing static EPR spectra with those acquired at increasing MAS frequencies. At 10 kHz MAS, the overall echo intensity decreases and the broad signal from P1 clusters (characterized by large anisotropic dipolar interactions) disappears completely. The resolved peaks also lose intensity, with the outer peaks exhibiting orientation-dependent lineshape distortion. As the MAS frequency increases to 25 kHz and then 37 kHz, the intensity of the outer peaks further diminishes until they are nearly absent while the central peak remains observable. Notably, in our previous work,^50^ complete dephasing of the powder peaks occurred at only 3 kHz MAS. The higher tolerance to MAS in the current study arises from the increased *B*_1_ mm-Wave field, which allows for shorter pulses and a shorter overall sequence duration (see Figure S1 for nutation results and *B*_1_ field strength measurements). The intensity changes and lineshape distortions of the resolved peaks are well-reproduced by numerical simulations (bottom panel, Figure 1a), using the same MATLAB-based code as in ref 50 but excluding P1 clusters.

To quantify MAS-induced dephasing, we measured two-pulse echo decays at 25 kHz MAS for the central (*ν*_mm‑Wave_ = 193.797 GHz) and right powder (*ν*_mm‑Wave_ = 193.880 GHz) peaks, as shown in Figure 1b. Both decays fit well to single exponentials with phase memory times (*T*_m_) of ∼0.39 μs and 0.16 μs, respectively. Comparing these to the static case (*T*_m_ = 3.5 μs) and 3 kHz MAS case (*T*_m_ = 0.9 μs) reveals that the dephasing accelerates with increased spinning speed, becoming nearly 10 times faster at 25 kHz than in the static case. Consequently, the electron spin signal decays within a few microseconds—much shorter than the rotor period even at 37 kHz (see Figure S2 and Table S1). Moreover, two-pulse MAS-EPR observed the electron spin coherence (transverse magnetization). But MAS-DNP relies on the transfer of polarization (longitudinal magnetization) of electron spins occurring on longer timescales. This fundamental difference limits the utility of two-pulse MAS-EPR for studying MAS-DNP mechanisms.

To observe longitudinal electron spin dynamics over these longer timescales, we chose to measure a three-pulse stimulated echo, whose decay is not only orders of magnitude slower than the *T*_m_ of P1 centers but also probes the decay of polarization of the electron spins. By varying the delay *t*_d_ between the second and third pulses (see inset in Figure 2a), we observed distinct echo decay behaviors under static and MAS conditions. While the static decay is monotonic and bi-exponential (Figure S3), the MAS decay exhibits periodic revivals (Figure 2a). In the different panels of this figure, the integrated three-pulse echo intensities are plotted for each *t*_d_ value at various MAS rates for the central EPR peak position (*m*_*I*_ = 0). Here, it can be observed that the echo is observable only for a few specific ranges of *t*_d_ values. For example, for the 25 kHz measurements (bottom panel in Figure 2a), the echo is observable for a few selected intervals of *t*_d_ around 40 μs, 80 μs, 120 μs, and so on, between which the echo intensity falls to zero. For the *t*_d_ values at which the echo intensity reaches a local maximum, we found that the pulse sequence is rotor-synchronized. In other words, the time interval between the beginning of the first and third pulses exactly equals an integer multiple of the rotor period.

**Figure 2:**
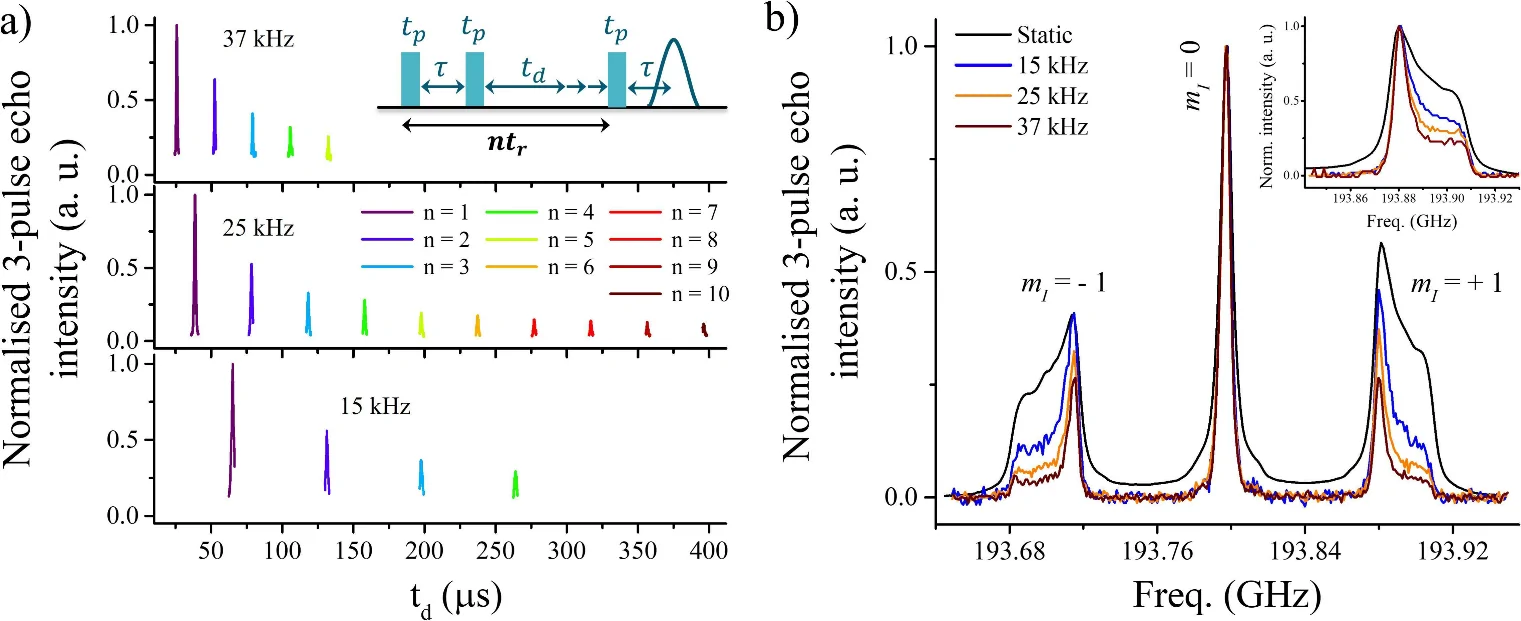
Three-pulse stimulated echo measurements at room temperature. (a) Echo intensity versus delay time *t*_d_ at (top) 37 kHz, (middle) 25 kHz, and (bottom) 15 kHz MAS. The inset shows the pulse sequence. Pulse sequence parameters: *t*_p_ = 400 ns, τ = 300 ns. *t*_d_ varies around integer multiples of the rotor period (*n* = 1 to 10). (b) Comparison of the frequency-swept three-pulse echo-detected spectra between static and the first rotor-period-synchronized MAS measurements. Inset: the same spectra normalized to the maximum of the powder pattern. Pulse sequence parameters for (b): *t*_p_ = 400 ns, τ = 300 ns, and *t*_d_ = 65.56, 38.9, and 25.93 μs for 15, 25, and 37 kHz MAS, respectively.

At 25 kHz, we recorded three-pulse stimulated echoes over 10 consecutive rotor periods (*n* = 1 to 10), reaching a maximum echo time of 400 μs (middlepanel of Figure 2a). In contrast, at 15 kHz (bottompanel) and 37 kHz (top panel), we could record three-pulse echoes over fewer rotor periods, reaching shorter overall echo times (Figure 2a). While shorter rotor periods at higher MAS frequencies allow more pulse sequence repetitions within the same time frame, the increase in MAS frequency also exacerbates MAS-induced spin dephasing during the pulses and the two τ periods. However, MAS-induced spin dephasing and shortening of rotor periods cannot entirely explain the differences in the longest achievable three-pulse echoes. This requires further investigation.

Using three-pulse rotor-synchronized experiments, we recorded frequency-swept EPR spectra at 15, 25, and 37 kHz MAS frequencies, corresponding to echo times of ∼67.37 μs, 40.70 μs, and 27.73 μs, respectively (Figure 2b). This approach recovers the powder lineshapes of the *m*_*I*_ = ±1 EPR lines, which were nearly invisible in the two-pulse experiment with an echo time of only 1.4 μs. This opens the possibility of observing spin dynamics at high MAS frequencies, which were previously inaccessible with two-pulse MAS-EPR due to rapid MAS-induced spin dephasing. However, the observed lineshapes differ from those in static EPR. To characterize this effect, we performed frequency-swept measurements around the powder peaks at various MAS frequencies (inset of Figure 2b). We observe a systematic increase in lineshape distortion with increasing MAS frequency. As detailed in our previous work,^50^ this distortion arises because for a given spin packet, the dephasing is weakest when the dipolar vector is perpendicular to the field, followed by the parallel orientation, and strongest in the center of the powder lineshape away from the canonical orientations.

To assess the role of synchronization precision, we recorded stimulated echo intensity while varying the pulse sequence duration around exact rotor periods (*n* = 1 to *n* = 10) in 50 ns steps. Figure 3a shows the echo intensity variation near the first rotor period (*n* = 1) at various MAS frequencies, measured at the central (*m*_*I*_ = 0) and powder (*m*_*I*_ = +1) EPR peak positions. As expected, the intensity becomes maximal when the pulse sequence is exactly synchronized with the rotor period (2*t*_p_ + τ + *t*_d_ = *t*_r_) and decreases with deviation from this condition. Generally, shorter echo times yield stronger intensities. This trend is observed for the *m*_*I*_ = 0 line, where echo intensity increases with MAS rate due to the shorter echo time. However, for the *m*_*I*_ = +1 line, the intensity increases only slightly between 10 and 15 kHz and then plateaus (Figure 3a). This is because MAS-induced spin dephasing, which increases with the MAS frequency, counteracts the benefit of shorter echo times for the hyperfine anisotropy-dominated *m*_*I*_ = +1 line. The simulated three-pulse echo intensities showed an excellent match with the experimental trends as shown in Figure 3b. Another effect visible in Figure 3a is the difference in width of the synchronization condition (matching tolerance) between the *m*_*I*_ = 0 and *m*_*I*_ = +1 lines at a given MAS frequency. The much narrower tolerance for the *m*_*I*_ = +1 line is another consequence of MAS-induced spin dephasing driven by hyperfine anisotropy.

**Figure 3:**
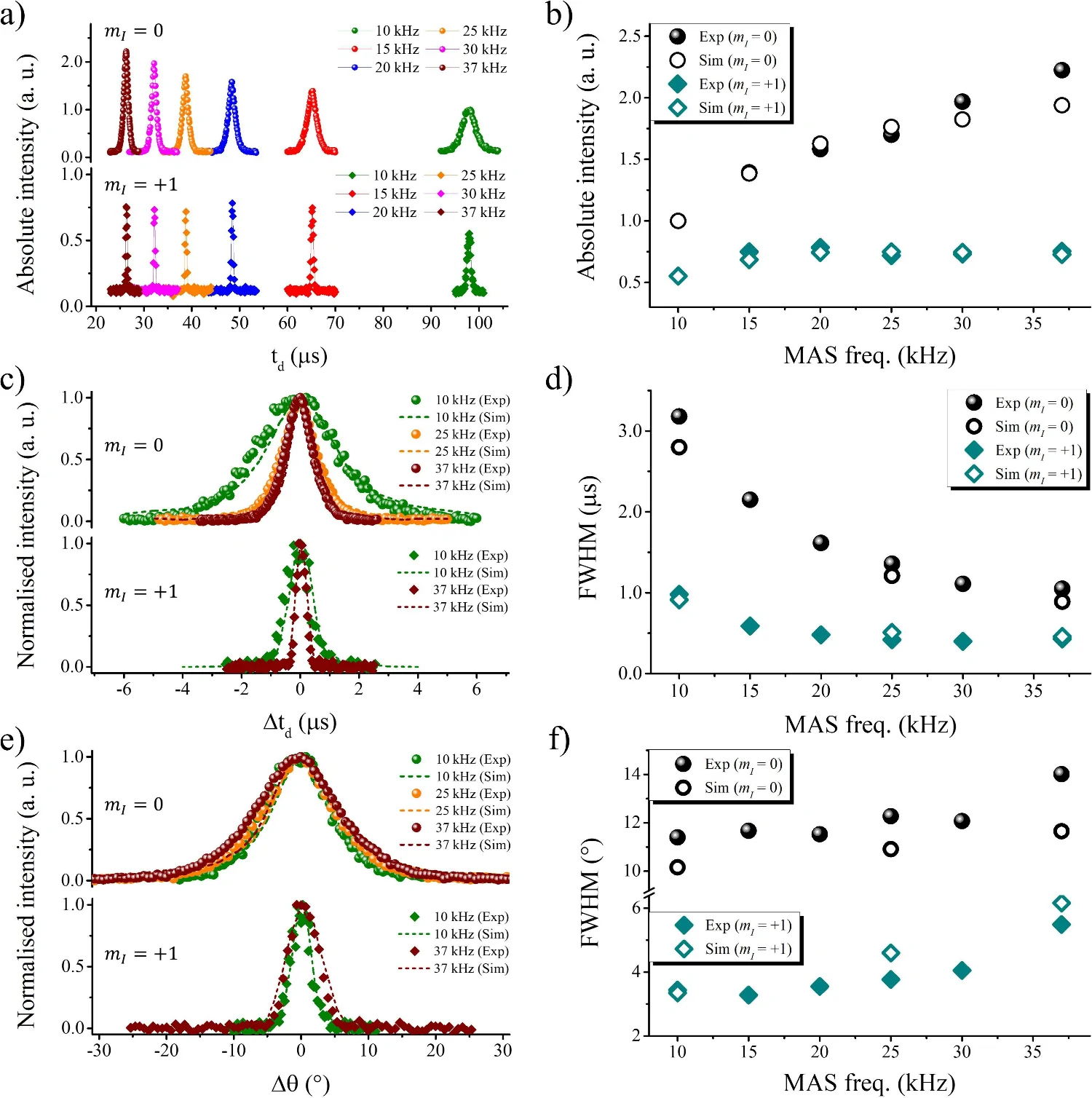
Role of rotor synchronization accuracy. (a) Three-pulse echo intensity versus delay time *t*_d_ around the first rotor period (*n* = 1) at various MAS frequencies, measured at the (top) central and (bottom) powder peak positions. (b) Rotor-synchronized echo intensity versus MAS rate for the *m*_*I*_ = 0 and *m*_*I*_ = +1 lines. Intensities are normalized to the maximum echo intensity of the *m*_*I*_ = 0 line at 10 kHz. (c) Normalized echo intensity versus offset Δ*t*_d_ = *t*_d_ – (*t*_r_ – 2*t*_p_ – τ) for 10, 25, and 37 kHz MAS rates at both peak positions. (d) Full width at half-maximum (FWHM) of the echo intensity profiles in (c) versus MAS rate for experimental and simulated data. (e) Three-pulse echo intensity versus phase offset Δθ for the *m*_*I*_ = 0 and *m*_*I*_ = +1 lines. (f) FWHM of the profiles in (e) versus MAS rate. In all panels, solid circles and squares denote experimental data, while dashed lines and empty squares represent simulations. Pulse parameters: *t*_p_ = 400 ns, τ = 300 ns; *t*_r_ is the rotor period.

Figure 3c overlays the synchronization profiles against the offset Δ*t*_d_ = *t*_d_ – (*t*_r_ – 2*t*_p_ – τ) for the *m*_*I*_ = 0 and *m*_*I*_ = +1 lines. In general, the profiles become narrower at higher MAS rates, indicating stricter synchronization requirements. This can be rationalized as follows: after the first pulse excites specific spin packets, their resonance frequencies shift due to reorientation during the τ period. At higher MAS rates, these spins move off-resonance more rapidly, reducing the efficiency of the second pulse in storing the polarization grating. Consequently, only when the third pulse is applied exactly one rotor period after the first pulse, all the stored gratings are fully refocused to form an echo. Thus, higher spinning frequencies demand tighter synchronization to maintain signal intensity, manifested as a reduced profile width with increasing MAS frequency (Figure 3d) in the experiment. However, the synchronization requirement also depends on the anisotropy of the EPR line, as the MAS-induced spin dephasing increases with overall anisotropy. Because of this, the profiles for the *m*_*I*_ = +1 line are significantly narrower than those for the *m*_*I*_ = 0 line (Figure 3c), reflecting the stricter synchronization requirements imposed by its higher hyperfine anisotropy. In addition, the difference in the profile widths between 10 and 37 kHz profiles is larger for the *m*_*I*_ = 0 line compared to the hyperfine anisotropy-dominated *m*_*I*_ = +1 line (Figure 3d).

To further corroborate this interpretation, we performed numerical simulations of the echo intensity dependence on rotor synchronization around the first rotor period at the central (*m*_*I*_ = 0) and powder (*m*_*I*_ = +1) peak positions (Figure 3c). Using the same pulse parameters as those in the experiments, the simulated profiles for each MAS frequency (dashed lines) agree well with the experimental results. Also, the experimental trends of reduced profile widths with increasing MAS frequency are well-reproduced in the simulations (Figure 3d; more details about the simulation can be found in the Supporting Information).

More subtle effects become visible when the echo profiles are plotted against the rotor phase offset: $\Delta \theta =360^{{}^{\circ}}\times \left(\frac{2t_{p}+\tau +t_{d}}{t_{r}}-1\right)$ . The exact synchronization (2*t*_p_ + τ + *t*_d_ = *t*_r_) corresponds to Δθ = 0 (a full 360° rotation), and the deviations from this condition are expressed as Δθ. When plotted against Δθ, the echo intensity for a given EPR line (fixed anisotropy) should depend only on the relative rotor orientation during the first and third pulses, effectively collapsing the data from different MAS frequencies onto a universal scale.

Indeed, the echo profiles for the central *m*_*I*_ = 0 line when plotted on this scale are nearly identical across MAS frequencies, as shown at the top in Figure 3e. Here, the intensity distribution follows a general master curve. A subtle, but consistent, increase in profile width with increasing MAS frequency is discussed later. Surprisingly, echo profiles plotted versus Δθ do not overlap for the *m*_*I*_ = +1 powder line (in the bottom of Figure 3e); the profile becomes wider (indicating a less stringent synchronization requirement) as MAS frequency increases. This trend is reproduced in simulations. This observation can be rationalized as follows: different spin packets span different frequency ranges over the rotor period and thus experience MAS dephasing to varying extents. Spin packets starting with the hyperfine tensor at canonical orientations dephase the most, while those starting closer to the magic angle span a smaller frequency range and dephase less. In other words, the two τ periods in the three-pulse sequence act as a filter, allowing spin packets with smaller effective anisotropies to contribute more to the stimulated echo. This filtering effect increases with MAS frequency. Consequently, at high MAS frequencies, spin packets with smaller anisotropies dominate the signal, resulting in a less stringent synchronization requirement and a broader profile. Note that this effect is visible only when the echo intensity profile is plotted on the rotor phase offset axis (Figure 3e). When plotted versus Δ*t*_d_ (Figure 3c), the profiles appear narrower at higher MAS frequencies because the ratio Δθ/Δ*t*_d_ increases with spinning rate.

We now return to the similar subtle trend observed for the central *m*_*I*_ = 0 line, which is nearly isotropic (shown at the top in Figure 3e). Note that both the FWHM and its increase with MAS frequency are more pronounced in the experiment than in the simulation. This discrepancy is attributed to unresolved anisotropic dipolar interactions with ^13^C nuclei and other electron spins, which were omitted from the simulations. This additional anisotropy causes variations in MAS-induced dephasing during the two τ periods of the three-pulse sequence. As a result, stimulated echoes at higher MAS frequencies consist of spin packets with smaller overall anisotropies (i.e., smaller dipolar interactions). These spins have less stringent refocusing requirements, leading to a slight broadening of the echo profile on the rotor phase scale (Figure 3f). This demonstrates that echo intensity versus Δθ profiles are highly sensitive to small anisotropic interactions—beyond the resolution of conventional echo-detected EPR.

In summary, we demonstrated 6.9 T magic angle spinning (MAS) pulse-EPR experiments at spinning frequencies up to 37 kHz—an order of magnitude faster than our previous work. This advancement enables rotor-synchronized three-pulse stimulated echo MAS-EPR experiments over multiple rotor periods, allowing stroboscopic probing of spin dynamics.

By varying the delay time *t*_d_ around the first rotor period, we characterized the time domain behavior of the spin packets. We found that synchronization requirements are stricter at higher MAS frequencies and depend strongly on the EPR line anisotropy, with larger anisotropies demanding tighter synchronization.

These results demonstrate that three-pulse rotor-synchronized experiments allow the observation of electron spin dynamics in powder samples at up to 37 kHz and on the 100 μs timescale—capabilities inaccessible with two-pulse MAS-EPR. We expect this methodology to provide critical insights into the role of electron spin dynamics in MAS-DNP mechanisms.

## Experimental Section

We procured PDA 657325400-D46 HPHT micro-diamond powder with an average particle size of 44 μm from Element Six, UK. Spin counting was performed on a Bruker Elexsys E500 CW X-band spectrometer to determine the P1 center concentration, which was found to be approximately 120 ppm. The diamond powder was filled into a 1.6 mm zirconia rotor manufactured by Phoenix NMR. The rotor was then loaded into the NMR-DNP probe prototype provided by Phoenix NMR. The probe was adapted for MAS-EPR experiments on the home-built DNP-EPR spectrometer at Tel-Aviv University.^53^ The hardware details of the MAS setup will be provided in a future publication. The spinning speed was controlled by a Bruker MAS II MAS control unit and was monitored on an oscilloscope. All of the measurements reported in this work were performed at room temperature.

The two-pulse MAS-EPR spectra were measured using a standard Hahn echo pulse sequence. The pulse lengths of the two pulses were kept the same at 400 ns (*t*_p_), with an interpulse delay of 300 ns (τ). A 16-step phase cycle was used for the pulses and detection as following: ϕ_1_ = [0°_4_, 90°_4_, 180°_4_, 270°_4_], ϕ_2_ = [0°, 90°, 180°, 270°]_4_, and ϕ_detection_ = ϕ_1_ – 2ϕ_2_. The EPR spectra were plotted by integrating echo intensity over the full width at half-maximum for each mm-Wave frequency.

For the three-pulse experiments, a stimulated echo sequence with equal pulse lengths (*t*_p_ = 400 ns) was employed. There was a fixed delay between the first and second pulses (τ = 300 ns). The *t*_d_ delay between the second and third pulses varied in each experiment; the values are given in the figures and in the figure captions. An eight-step phase cycle was used to record the stimulated echoes with the following phases: ϕ_1_ = [0°_4_, 180°_4_], ϕ_2_ = [0°_2_, 180°_2_]_2_, ϕ_3_ = [0°, 180°]_4_, and ϕ_detection_ = ϕ_1_ + ϕ_2_ + ϕ_3_. In the case of frequency-swept three-pulse experiments, the mm-Wave frequency was stepped, and the integrated stimulated echo intensity was plotted for each mm-Wave frequency to obtain a three-pulse MAS-EPR spectrum.

## Supplementary Material



## Notes

Static three-pulse nutation, phase memory times at different MAS rates, static stimulated echo decay, and MAS-EPR simulation methodology. (PDF)
