# Supplementary material for: Observation of Electron Spin Dynamics on Hundreds-of-Microseconds Timescales in High-Field Magic Angle Spinning EPR
Source: J Phys Chem Lett. 2026 Sep 19;17(39):11017–23. doi: 10.1021/acs.jpclett.6c02108 (PMC13637070; doi:10.1021/acs.jpclett.6c02108)
Supplement: Supplementary file 1 [file jz6c02108_si_002.pdf]

# Supplementary Information: Observation of Electron Spin Dynamics on Hundreds-of-Microseconds Timescales in High-Field Magic Angle Spinning EPR

Sudipta Khamrui,† Orit Nir-Arad,† Frederic Mentink-Vigier,‡ Alexander B. Fialkov,† Nurit Manukovsky,† and Ilia Kaminker*,†

†*School of Chemistry, Tel Aviv University, 6997801 Tel Aviv, Israel.*

‡*National High Magnetic Field Laboratory, Florida State University, Tallahassee, FL 32310, USA, and Department of Chemistry and Biochemistry, Florida State University, Tallahassee, Florida 32306, USA.*

E-mail: iliakam@tauex.tau.ac.il

## Three-pulse nutation measurements

Here, we present the static nutation data measured at the central peak position of the P1 EPR spectrum as shown in **Figure S1**. It can be seen that the first minimum of the nutation curve occurs around 1.2 $\mu s$ in this work, which is approximately two times shorter than the value achieved in our previous setup. This corresponds to a nutation frequency of $\sim$ 416 kHz.

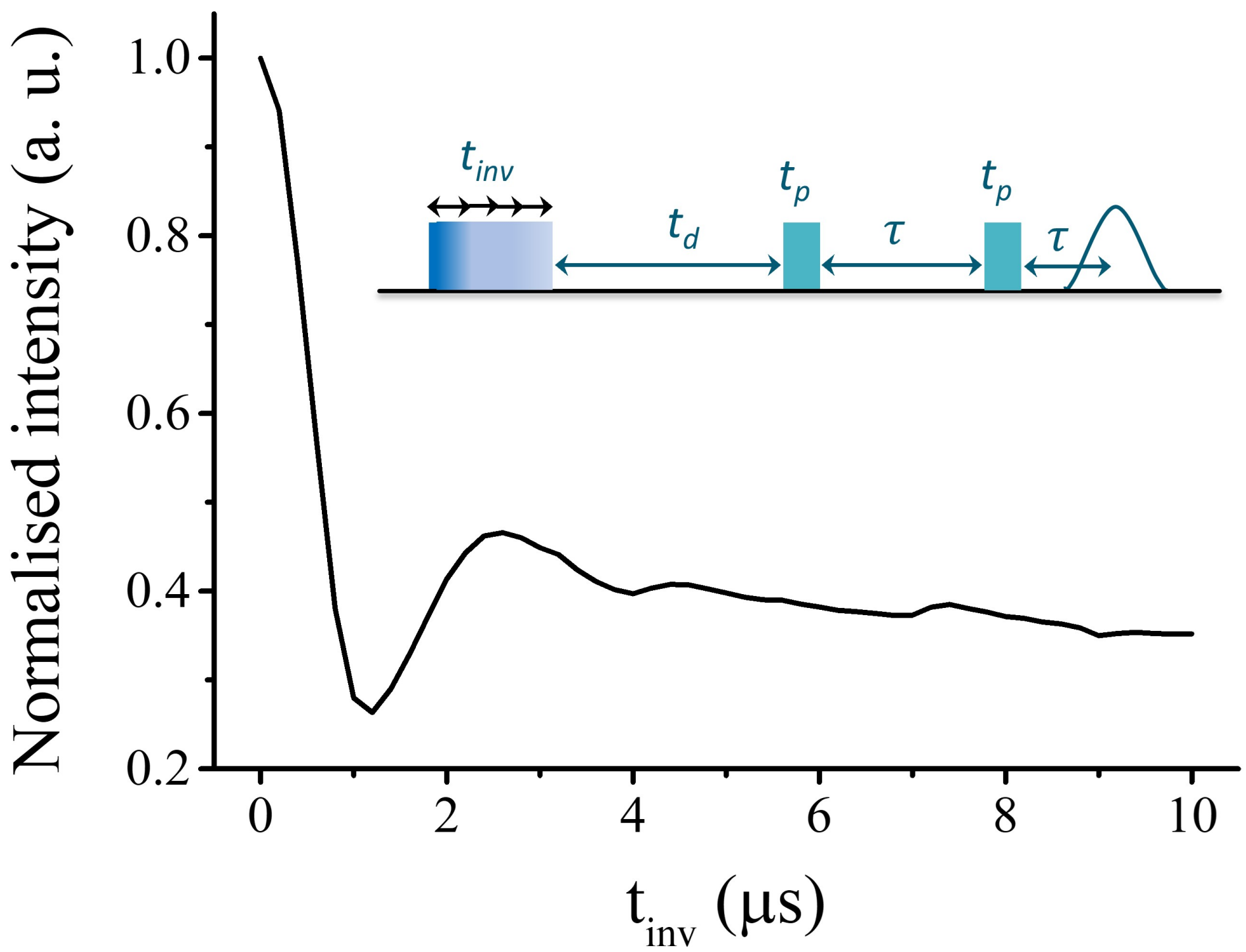


Figure S1: Static three-pulse nutation measured at the central peak of P1 spectrum. Pulse parameters: $t_{inv} = 0$ to $10\ \mu s$, increased in steps of $50\ ns$; $t_p = 650\ ns$; $\tau = 500\ ns$, and $t_d = 10\ \mu s$.

## Phase memory time measurements at various MAS rates

**Figure S2** shows the comparison of the phase memory times ($T_m$) of the central ($m_I = 0$) and powder ($m_I = +\,1$) spin packets measured under static conditions and at different MAS frequencies. From static conditions to 3 kHz MAS, the dephasing time has decreased to approximately $\frac{1}{4}$ of the static value. However, at higher MAS frequencies, it has a moderate dependence on the spinning rate. The values of the phase memory times are listed in **Table S1**. The echo decays were fitted with single exponential decay curves. It can be observed that the faster the MAS, the stronger the MAS-induced spin dephasing. This means that, regardless of how fast we spin the sample, we can measure spin dynamics only on a timescale of a few microseconds using a two-pulse echo sequence.

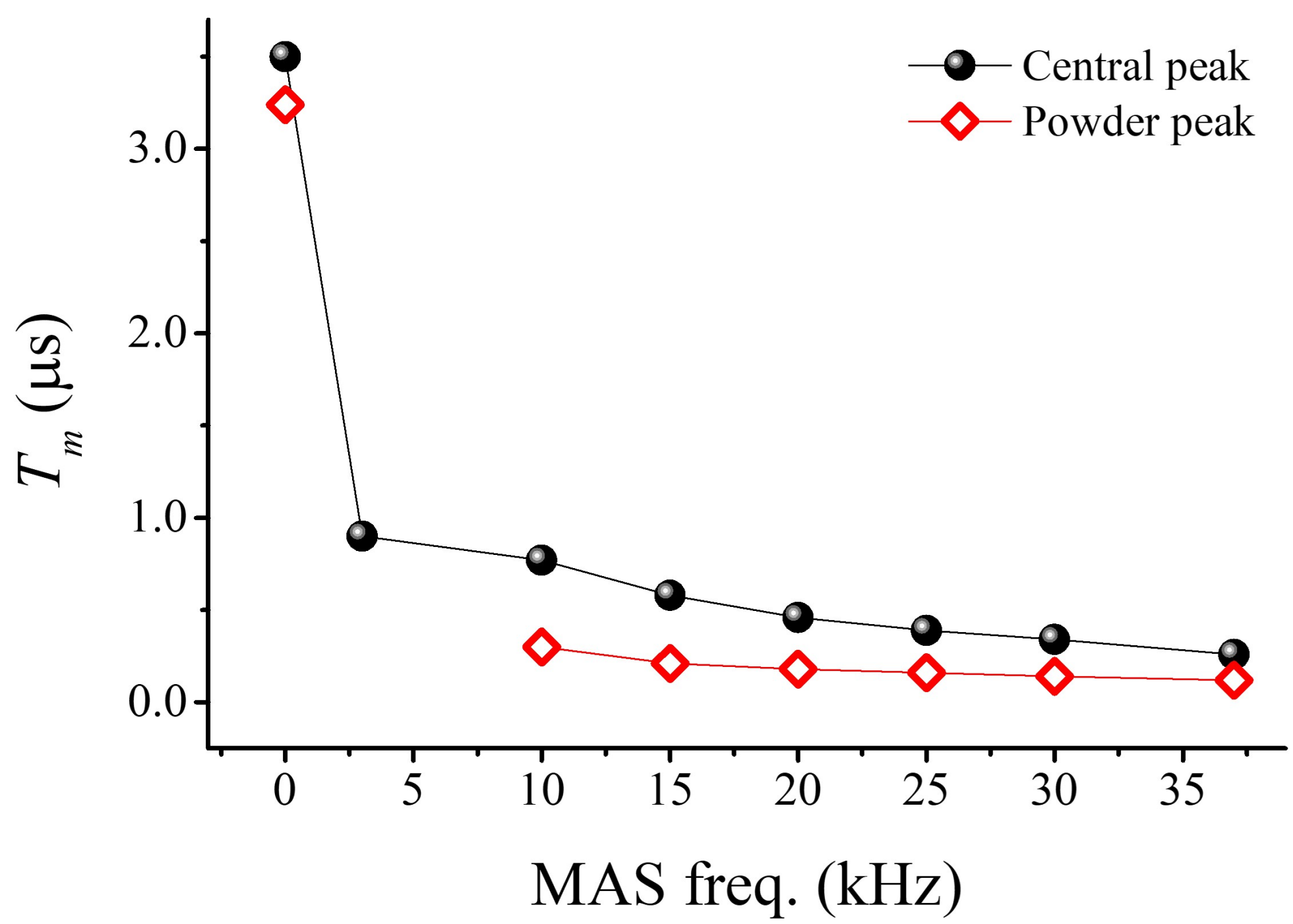


Figure S2: Phase memory time variation of the central and powder spin packets with MAS frequency. The positions of the echo decay measurements are the same as marked by the black and red down arrows in Figure 1(a) of the main text.

Table S1: List of phase memory times ($T_m$) measured at the central ($m_I = 0$) peak and at the maximum-intensity position of the right powder ($m_I = +1$) peak under static conditions and at different MAS frequencies. Both the static and MAS echo decays could be fitted with single exponential decay curves. The phase memory times were calculated from the decay constants obtained from those fits.

| MAS frequency (kHz) | $T_m$ (in $\mu s$) for the central spin packets ($m_I = 0$) | $T_m$ (in $\mu s$) for the powder spin packets ($m_I = +1$) |
|---|---|---|
| 0 | 3.50 | 3.24 |
| 3 | 0.90 | - |
| 10 | 0.77 | 0.30 |
| 15 | 0.58 | 0.21 |
| 20 | 0.46 | 0.18 |
| 25 | 0.39 | 0.16 |
| 30 | 0.34 | 0.14 |
| 37 | 0.26 | 0.12 |

## Static three-pulse stimulated echo decay

**Figure S3** shows the static three-pulse stimulated echo decay at the central peak of the P1 EPR spectrum. A significant non-zero echo intensity is observed up to $\sim 500\,\mu s$, which allows stimulated echo measurements over a longer delay time scale. The experimental data could be fitted to a linear combination of an exponential and a stretched exponential decay curves. The time constants of the fit were as follows: $t_1 \sim 100\,\mu s$ (stretch factor = 0.7; weight $\sim$ 0.26) and $t_2 \sim 9.67\,\mu s$ (weight $\sim$ 0.74).

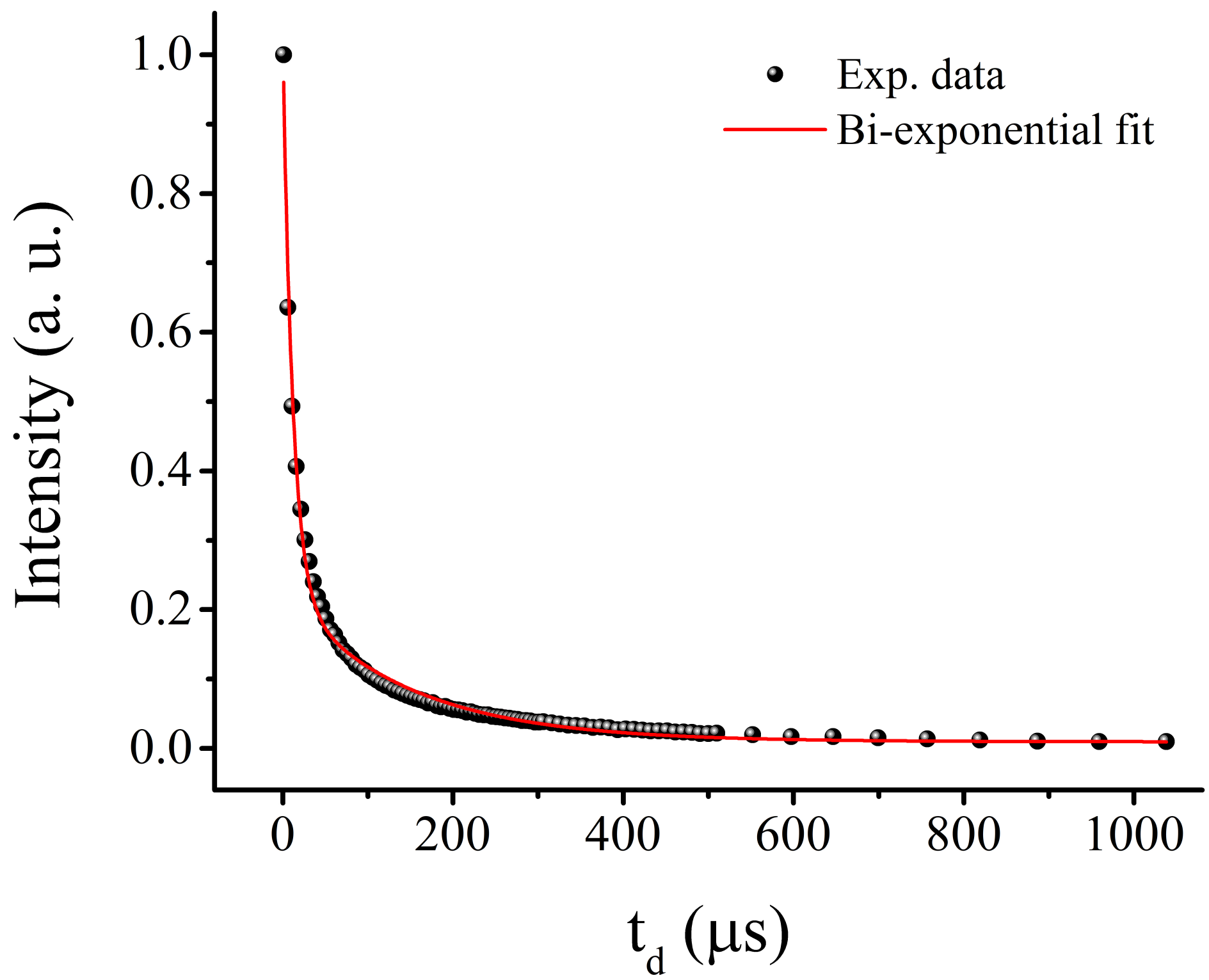


Figure S3: Three-pulse stimulated echo decay measurement at the central peak position under static conditions.

## MAS-EPR Simulation

We performed the MAS-EPR simulations using an in-house MATLAB code. The methodology is the same as reported in our previous MAS report.[1] The only difference is the use of three-pulse instead of two-pulse sequence. We considered isolated P1 centres, where an unpaired electron ($S = \frac{1}{2}$) is coupled to a $^{14}N$ nucleus ($I = 1$) through hyperfine interaction. In the simulation, we did not consider electron-electron couplings for computational simplicity. Also, we neglected the $^{14}N$ quadrupole coupling. The static Hamiltonian of these isolated P1 centres in the electron's rotating frame is the following:

$$\hat{H} = [\frac{g\mu_B B_0}{\hbar} - \omega_{mw}]\hat{S}_z + \hat{S}_x\omega_1 cos(\phi) + \hat{S}_y\omega_1 sin(\phi) + A_z\hat{S}_z\hat{I}_z \quad (1)$$

The secular part of the hyperfine coupling can further be written as:

$$A_z = 2\pi(A_{\parallel}\, cos^2\theta + A_{\perp}\, sin^2\theta) \quad (2)$$

In these equations, $g$ is the Lande $g$-tensor value; $\omega_{mw}$ is the mm-Wave frequency; $\omega_1$ the nutation frequency; $\phi$ is phase of the mm-Wave pulses in the rotating frame; $\hat{S}_x$, $\hat{S}_y$ and $\hat{S}_z$ are the electron spin operators; $\hat{I}_z$ is the nuclear spin operator; $\theta$ is the angle between the magnetic field $B_0$ and the direction of the $e -^{14} N$ dipolar interaction; $A_{\parallel}$ and $A_{\perp}$ are the parallel and perpendicular components of the $^{14}N$ hyperfine interaction.

In the simulation, we considered the same pulse parameters as used in the experiment: $t_p$ = 400 $ns$; $\tau$ = 300 $ns$; while varying the $t_d$. The initial values of the interaction parameters were the following as obtained from the static experiments: nutation frequeny, $\omega_1 = 2\pi\times0.416$ MHz; the Lande g-tensor, $g_{\perp}$ = 2.00220 and $g_{\parallel}$ = 2.00218; the hyperfine-tensor, $A_{\perp}$ = 81.3 MHz and $A_{\parallel}$ = 114 MHz. Under MAS, the Lande $g(t)$, and the hyperfine coupling $A_z(t)$ terms become time-dependent. The time steps in the simulation were set to $\delta t = \frac{1}{2^{14}\times\nu_r}$. A three-angular Lebedev quadrature grid of rank 41 was used for powder averaging.[2] The

electron spin relaxation times were fixed to $T_1 = 100\ \mu s$ and $T_2 = 4\ \mu s$, which are close to the experimentally measured static values for the powder sample.

## References

(1) Nir-Arad, O.; Fialkov, A. B.; Shlomi, D. H.; Manukovsky, N.; Mentink-Vigier, F.; Kaminker, I. High-field pulsed EPR spectroscopy under magic angle spinning. *Science Advances* **2024**, *10*, eadq6073.

(2) Lebedev, V. I. Quadratures on a sphere. *USSR Computational Mathematics and Mathematical Physics* **1976**, *16*, 10–24.
